# Co-Production of Care Plans to Improve Safety on High-Dependency Rehabilitation Psychiatric Wards

**DOI:** 10.1192/bjo.2025.10391

**Published:** 2025-06-20

**Authors:** Omer Malik, Angela Misra

**Affiliations:** Cygnet Healthcare, London, United Kingdom

## Abstract

**Aims:** Care plans are the cornerstone of Rehabilitation Psychiatry. These were not being completed collaboratively with patients during monthly ward rounds, leading to impaired communication, lack of patient involvement in risk reduction strategies, and frustration. This contributed to volatility and increased risk incidents.

This Quality Improvement Project aimed to Co-produce Care Plans with staff and patients with primary outcomes relating to reducing risk incidents and secondary outcomes aiming to improve Staff and Patient engagement with Care Plans.

**Methods:** Baseline questionnaires (Likert scale and open-ended questions) were conducted with clinical staff and patients to assess care plan satisfaction. Feedback revealed concerns about care plan length (average of 40–60 pages), user-friendliness, appropriateness and engagement with patient/carer views not captured conspicuously.

This feedback was discussed with the Senior Management Team and care plan survey participants. A report with graphs was sent to Maple ward staff and stakeholders and later presented at the National Steering Rehabilitation Cygnet group meeting.

A new care plan template was co-produced in two focused group meetings (8 paged). The ward round format was changed to being care plan-based, aligned with Cygnet’s rehabilitation standards. A multimedia screen was purchased to support collaborative care plan completion. The new care plan featured columns for verbatim patient views, relevant discipline feedback, goals, and evaluations. The rows included sections for Treatment (Psychology, Occupational Therapy, Nursing, Social Needs, and Medical), Safety (Risk Management Plan), Recovery (Discharge Planning), Physical Health, Well-being (Spiritual, Cultural, Protective Factors), and Monthly Patient Progress Feedback.

**Results:** Primary outcomes showed a substantial reduction in physical restraints (65.5%), rapid tranquillisations (90%), and physical aggression (35%). However, verbal aggression incidents increased by 311%, though they did not escalate to restraints or tranquillisations, indicating improved ward safety.

The project led to a significant positive impact: 100% of patients now read their clear, concise care plans. Secondary outcomes showed an increase in patient satisfaction with care plans (57%), staff views on care plan effectiveness (60%), and a reduction in care plan length (62%).

**Conclusion:** Patients, carers, families, social workers, and advocates actively collaborated with multidisciplinary staff and Community Mental Health Teams to achieve shared goals and provide real-time feedback. To support co-production initiatives, infrastructure must be in place, and processes should be streamlined to ensure efficient embedding of quality improvement learning, including policy updates.

Given the successful outcomes of the pilot, the project is being considered for national rollout across Cygnet Healthcare’s rehabilitation wards.

